# The impact of government actions and risk perception on the promotion of self-protective behaviors during the COVID-19 pandemic

**DOI:** 10.1371/journal.pone.0284433

**Published:** 2023-04-17

**Authors:** Javier Alvarez-Galvez, Andreas Anastasiou, Demetris Lamnisos, Marios Constantinou, Christiana Nicolaou, Savvas Papacostas, Vasilis S. Vasiliou, Louise McHugh, Jelena Lubenko, Francisco J. Ruiz, Marisa Paez-Blarrina, Francisco Montesinos, Sonsoles Valdivia-Salas, Rhonda M. Merwin, Maria Karekla, Andrew T. Gloster, Angelos P. Kassianos

**Affiliations:** 1 Department of Biomedicine, Biotechnology and Public Health, University of Cadiz, Cadiz, Spain; 2 Department of Mathematics and Statistics, University of Cyprus, Nicosia, Cyprus; 3 Department of Health Sciences, European University of Cyprus, Nicosia, Cyprus; 4 Department of Psychology, University of Nicosia, Nicosia, Cyprus; 5 Department of Nursing, School of Health Sciences, Cyprus University of Technology, Limassol, Cyprus; 6 Cyprus Institute of Neurology and Genetics, Nicosia, Cyprus; 7 Nuffield Department of Orthopaedics, Rheumatology and Musculoskeletal Sciences (NDORMS), University of Oxford, Oxford, United Kingdom; 8 School of Psychology, University College Dublin, Dublin, Ireland; 9 Psychological Laboratory, Faculty of Public Health and Social Welfare, Riga Stradings University, Riga, Latvia; 10 Fundación Universitaria Konrad Lorenz, Bogotá, Colombia; 11 Universidad Europea de Madrid, Madrid, Spain; 12 Instituto ACT, Madrid, Spain; 13 Universidad de Zaragoza, Zaragoza, Spain; 14 Department of Psychiatry and Behavioral Science, Duke University, Durham, North Carolina, United States of America; 15 Department of Psychology, University of Cyprus, Nicosia, Cyprus; 16 Division of Clinical Psychology & Intervention Science, Department of Psychology, University of Basel, Basel, Switzerland; 17 Department of Applied Health Research, University College London, London, United Kingdom; Queen Mary University of London, UNITED KINGDOM

## Abstract

**Introduction:**

We aim to understand the factors that drive citizens of different countries to adhere to recommended self-protective behaviors during the COVID-19 pandemic.

**Methods:**

Survey data was obtained through the COVID-19 Impact project. We selected countries that presented a sufficiently complete time series and a statistically relevant sample for running the analysis: Cyprus, Germany, Greece, Ireland, Latvia, Spain, Switzerland, the United Kingdom, and the United States of America. To identify country-specific differences in self-protective behaviors, we used previous evidence and change-point detection analysis to establish variations across participating countries whose effect was then assessed by means of interrupted series analysis.

**Results:**

A high level of compliance with health and governmental authorities’ recommendations were generally observed in all included countries. The level of stress decreased near the period when countries such as Cyprus, Greece or the United Kingdom relaxed their prevention behavior recommendations. However, this relaxation of behaviors did not occur in countries such as Germany, Ireland, or the United States. As observed in the change-point detection analysis, when the daily number of recorded COVID-19 cases decreased, people relaxed their protective behaviors (Cyprus, Greece, Ireland), although the opposite trend was observed in Switzerland.

**Discussion:**

COVID-19 self-protective behaviors were heterogeneous across countries examined. Our findings show that there is probably no single winning strategy for exiting future health crises, as similar interventions, aimed to promote self-protective behaviors, may be received differently depending on the specific population groups and on the particular geographical context in which they are implemented.

## Background

The COVID-19 pandemic has highlighted the challenges governments and health agencies have faced to protect the population during this health emergency [1]. As recent studies have shown, part of the success of policy actions is related to the appropriate strategies to manage the course of the disease and mitigate possible risk behaviors [2–4]. The selection of appropriate governmental strategies to manage the course of the disease and mitigate possible risk behaviors is considered to be a fundamental element in the control of the COVID-19 pandemic. While developing pharmacological interventions for COVID-19 spread prevention and management, the mitigation of the disease depends on non-pharmacological interventions and behaviors people can follow, especially when self-protective behaviors are known to be related to threat perceptions, social and cultural norms, stress, and coping [5].

In addition, how people perceive their risk is known to drive self-protective behaviors in early interventions during large-scale pandemics. The COVID-19 Social Study in the UK has revealed some evidence which helps us to understand population compliance. For example, findings from the study indicated via self-report data that mask wearing was the most complied with behavior and social distancing the least [6]. Another study has supported similar findings controlling for COVID-19 caseload and containment policies [7]. Evidence from the US has also shed light on personal characteristics that shape behavior such as partisanship [8] whilst recent evidence from Vietnam highlighted that geographic location, culture and behaviors exhibited in social media are important contributors on how people determine their risk for COVID-19 [9, 10].

Perceptions of threat and severity constitute common reactions during pandemics; such reactions can be contagious among individuals [11, 12]. The impact of fear on self-protective behaviors depends on individuals’ perceptions [13]. An individual’s fear and behaviors exhibited that result from perceptions of threat can escalate if they perceive governmental crisis response as inconsistent, incompetent, unfair, subjective, non-empathetic and insincere [14]. Individuals’ perceptions of fear can be exaggerated by evidence of contagion and mortality but how these perceptions influence self-protective behaviors is currently unknown or only partly known.

The Protection Motivation Theory [15] ascertains that fear messages trigger individual appraisal of a potential threat. In cases when the threat appraisal prevails over coping appraisal then people demonstrate maladaptive responses such as denial, whereas when the coping response prevails (such as perceived self-efficacy) then people demonstrate protection motivation. A meta-analysis has demonstrated that increases in perceptions of threat severity, threat vulnerability, response efficacy, and self-efficacy facilitate adaptive behaviors [16]. If this also applies to how people react to pandemic threats such as COVID-19, then intervention programs can focus on lowering maladaptive response to potential threats and foster adaptive behaviors (i.e., protective behaviors such as mask wearing or keeping physical distancing).

Recent evidence suggests that self-protective behaviors from COVID-19 are associated with individuals’ beliefs of efficacy in promoting self-protective behaviors and how much they value health [17] their perceived credibility of information provided [18] their levels of psychologically flexible in the face of threats, their prosociality [19], perceived threat/illness [20] and perceptions of risk [21]. More recent studies have started examining fear and perceptions of threat, triangulating self-reported cross-sectional data with more objective population health indicators. In addition, public-health messages ought to be acceptable, credible, and trustworthy, to increase adherence to self-protective behaviors, consequently increasing the public’s understanding and tackling perceptions of the threat [22].

To date, the impact of government interventions aimed to control the pandemic on people’s self-protective behaviors and the role of their risk perceptions remain unclear. Many studies examining these hypotheses use country-specific data while evidence points towards adherence to protective measures being strongly influenced by contextual factors [23, 24]. Thus we sought to use multi-country data to reinforce and triangulate self-report data with more objective indicators of fear such as the retrospective collection of the daily number of newly diagnosed cases or the incidences of deaths. For this research, we selected case studies according to the availability of data from the "COVID-19 Impact” project, to understand how people from different countries adhere to recommended self-protective behaviors during the COVID-19 pandemic. For this aim, we hypothesized that governments’ responses aiming to control the spread of the pandemic have been able to–at least partially–reduce risk behaviors (hypothesis 1), but we also hypothesize that, during health emergencies such as the COVID-19 pandemic, perceptions of threat and resulting fear may be a key coping factor in adhering to self-protective behaviors (hypothesis 2).

## Methods

### Data and countries

This is a secondary data analysis utilizing data from the COVID-19 Impact project survey, a population based cross-sectional study that aimed to understand factors that can impact people’s protective behaviors during the early stages of the COVID-19 pandemic. This dataset includes information from adult participants (≥18 years of age) from 78 countries. The data were collected for two months between the 7th of April and the 7th of June 2020. At the time of data collection, most participating countries had declared a state of emergency for COVID-19 and were on lockdown. Additional information on the project can be found in Gloster et al. [25].

In order to facilitate the comparison between the different units of analysis, we selected countries that presented a sufficiently complete time series (at least 30 days of data per country) and a statistically relevant sample for the implementation of the analysis. The countries selected that met these criteria as case studies were the following: Cyprus (N = 957), Germany (N = 279), Greece (N = 270), Ireland (N = 414), Latvia (N = 1285), Spain (N = 296), Switzerland (N = 550), United Kingdom (N = 100), and United States of America (N = 268).

### Variables of study

We focused on three main variables that captured COVID-19 self-protective behaviors: hand washing, isolation, and social distancing. Participants were asked to respond on a 10-point Likert scale ranging from never (0) to all the time (10) whether they followed these self-protective behavior recommendations. Using these three indicators, a new outcome variable measuring overall adherence to (recommended) COVID-19 self-protective behaviors was constructed. The response variable was obtained as a mean score of the aforementioned three indicators, having values between 0 and 10, where 0 = *minimum adherence* and 10 = *maximum adherence* to the COVID-19 self-protective behaviors. The reason for choosing to pool these three variables was due to the need to reduce possible variations due to sample size at different time points, while at the same time obtaining an overall measure of compliance with internationally established behavioral recommendations during the COVID-19 pandemic.

To capture variations in adherence to protective behaviors measures during the COVID-19 pandemic along the different countries under study, we used a governmental measures’ stringency index that was extracted through the Oxford COVID-19 Government Response Tracker (OxCGRT), which is a continuously updated dataset that addresses the need for comparable information on policy measures during the pandemic [1]. This dataset contains information about government policies related to closure and containment, health, and economic policy for more than 180 countries from the 1^st^ of January 2020. The stringency index is a composite measure based on nine response indicators: (1) school closures, (2) workplace closures, (3) cancellation of public events, (4) restriction on gatherings, (5) public transport closures, (6) stay at home requirements, (7) restriction on movements, (8) international travel controls, and (9) public information campaigns. These indicators are rescaled to a value from 0 to 100 (where 100 = strictest).

Additionally, for our second study hypothesis, we considered the time points at which the numbers of COVID-19 confirmed cases peaked during the analysis period. In this way, we aimed to analyze the impact of the course of the pandemic on possible variations in health behaviors and, indirectly, on general adherence to health and national authorities’ norms.

### Statistical analysis

To study the factors that lead citizens of different countries to follow the rules during the COVID-19 pandemic, we used interrupted time series analysis (ITSA) [26–29]. The ITSA is an extension of the Difference-in-Differences (DiD) design that compares trends in an outcome over multiple pre- and post- time-period measures, allowing for an interruption (i.e., a discontinuity) in both control and exposure average rates during the period under study. This technique was used to examine the impact of governmental actions on the control of COVID self-protective behaviors (i.e., the intervention) during the COVID-19 pandemic. As an advantage, in contrast to other statistical techniques, ITSA works with data sequences with missing information, but also allows exploration of the impact of contextual events which might explain changes in data trends (in our case, the effect of governmental actions and/or the effect of disease waves/peaks on people’s protective behaviors).

The standard ITSA model [22–25] can be described as:

$$Y_{t}=\beta_{0}+\beta_{1}T_{t}+\beta_{2}X_{t}+\beta_{3}X_{t}T_{t}+\epsilon_{t}$$

In this model, *Y*_*t*_ represents the outcome variable measured at each equally spaced *t* (time), *T*_*t*_ is the time elapsed from the initiation of the study, *X*_*t*_ is a binary variable representing the intervention time we are interested in (i.e., a change or interruption in the time series), and finally, an interaction term which is represented by *X*_*t*_*T*_*t*_. In this standard model, *β*_*0*_ is the constant (or intercept) of the response variable, *β*_*1*_ represents the slope of the outcome variable prior to the inclusion of the intervention, *β*_*2*_ represents the change in the level of the response variable after the intervention, and *β*_*3*_ is the difference between pre- and post-intervention slopes of the outcome variable. Thus, the fundamental objective of the ITSA is to look for significant *p-values* in either *β*_*2*_ to identify any subsequent change in self-protective behaviors, or in *β*_*3*_ in order to identify a treatment effect along the time of study [23, 25].

In terms of data acquisition, change-point detection analysis (CPDA) is split into two main categories; *a-posteriori* detection where the data are already obtained prior to the analysis, and *online* detection where the observations arrive sequentially at present. In our study, the focus is on a-posteriori change-point detection being applied to two main pillars of our data; firstly, on the daily number of new COVID-19 cases and deaths, and secondly, on the COVID-19 self-protective behavior variables as described in the Methods section. The model that we work on is

$$X_{t}=f_{t}+\sigma \epsilon_{t},\;t=1,2,\ldots ,T$$ (1)

where *T* is the length of the given data sequence, *X*_*t*_ are the observed data, while *f*_*t*_ is an unknown one-dimensional, piecewise-constant signal with abrupt changes in the mean. The number, *N*, of the change-points as well as their locations *r*_1_, *r*_2_, …, *r*_*N*_ are unknown and the aim here is to estimate them. The random variables ϵ_*t*_ have mean zero and variance one, while *σ* > 0

Detecting changes in the mean or the slope of the data sequence *X*_*t*_ as expressed in Eq (1) allows us to separate the given data sequence into homogeneous segments, which leads to more flexible models. In addition, the advantages of such change detections are split into two main categories; interpretation and forecasting. Interpretation comes naturally since the detected changes are often connected with life events that took place near the estimated change-point location. Associating the results with such real-life phenomena can easily lead to a better understanding of the behavior of the data at hand.

The Isolate-Detect (ID) methodology [30] is employed in order to detect changes based on the model given in Eq (1). The basic idea in ID is that for an observed data sequence of length *T* and with *λ*_*t*_ a positive constant, ID first creates two ordered sets of *K* = *T/λ*_*t*_ right- and left-expanding intervals. The *j*^*th*^ right-expanding interval is *R_j_* = [1, *min*{*jλ*_*t*_,*T*}] while the *j*^*th*^ left-expanding interval is *L*_*j*_ = [*max*{1, *T*—*jλ*_*T*_ + 1},*T*]. We collect these intervals in the ordered set *S*_*RL*_ = {*R*_1_, *L*_1_, *R*_2_, *L*_2_, …, *R*_*K*_, *L*_*K*_}. For a suitably chosen contrast function (details on its choice are given in Section 3.2 of Anastasiou and Fryzlewicz [30]), ID identifies the point, $\tilde{b}$, with the maximum contrast function value in *R*_1_. If its value exceeds a predefined threshold, which has been explicitly derived in [26], then $\tilde{b}$ is taken as a change-point. If not, then the next interval in *S*_*RL*_ is tested. Upon detection, ID makes a new start from the end-point (or start-point) of the right- (or left-) expanding interval where the detection occurred. When compared to the state-of-the-art competitors in the literature, in simulations carried out in [26] ID lies in the top 10% (in terms of the accurate estimation of the number and the location of the change-points) of the best methods; in most scenarios it is the best method overall. In addition, ID attains a low computational cost, and it can accurately analyse signals of tens of thousands with thousands of change-points in less than a second. Isolate-Detect is implemented in the R packages **IDetect** and **breakfast** available from CRAN.

In this article, our focus is on the detection of changes in the mean or the trend of the unknown signal *f*_*t*_. In the former case of changes in the mean, the signal is assumed to be piecewise-constant (Fig 2), while in the latter case, where we seek changes in the trend, then *f*_*t*_ is assumed to be continuous and piecewise-linear (Fig 3).

### Ethics statement

For this study, ethics approval was acquired from the Cyprus National Bioethics Committee (ref.: EEBK EΠ 2020.01.60) followed by site approvals from different research groups involved in data collection. All participants of the survey provided written informed consent prior to completing the survey (i.e., computer based, by clicking “yes” in the online system).

## Results

### Descriptive analysis of self-protective behaviors

**Fig 1:**
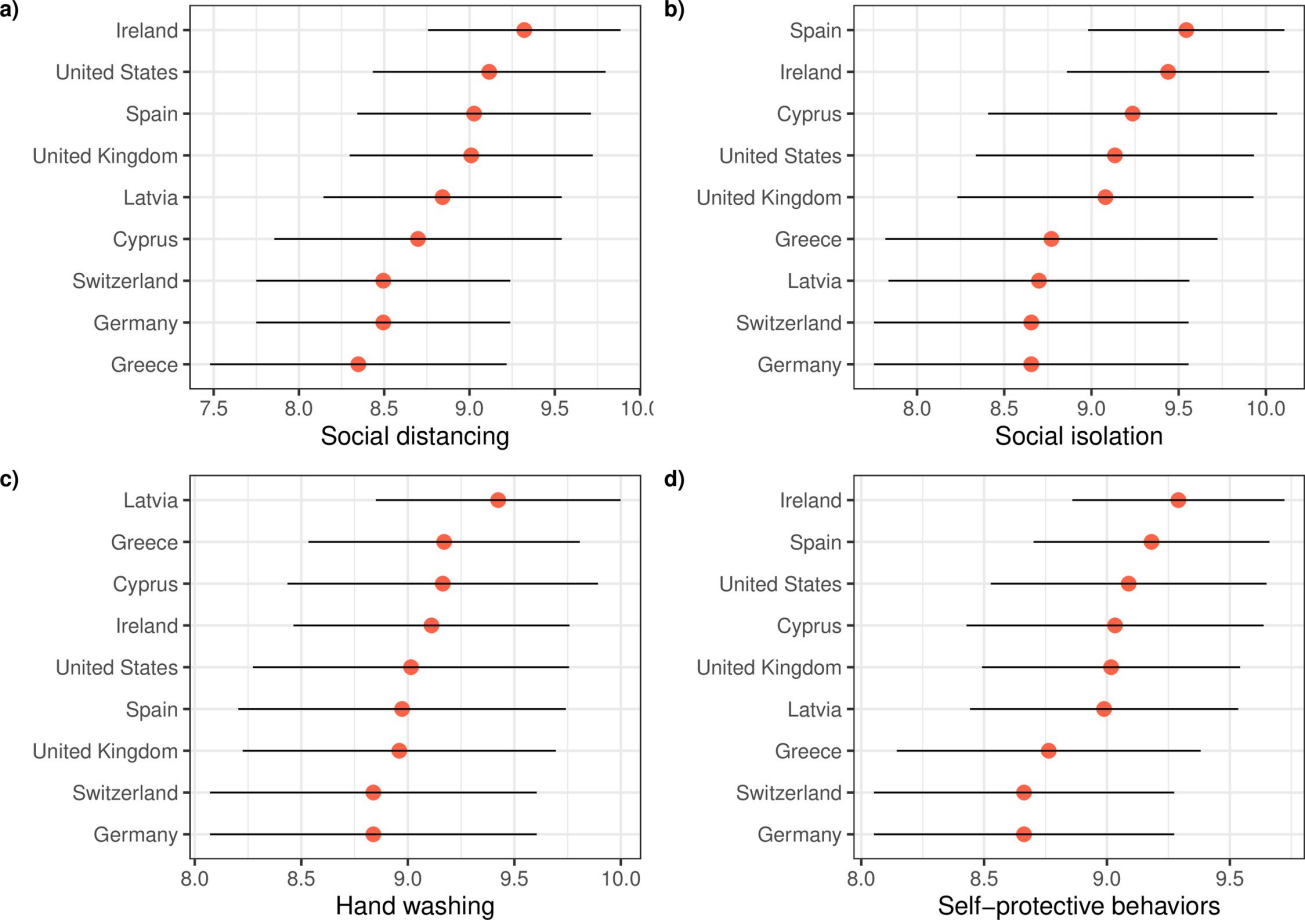
Mean and standard deviations for the variables a) social distancing, b) social isolation, c) hand washing, and d) protective behaviors in nine countries. The sample sizes for the different countries were as follows: Cyprus (N = 957), Germany (N = 279), Greece (N = 270), Ireland (N = 414), Latvia (N = 1285), Spain (N = 296), Switzerland (N = 550), United Kingdom (N = 100), and United States of America (N = 268).

A high level of adherence with the recommendations of health and governmental authorities was observed in all the countries under analysis (Fig 1). The recommended (or, given the case, mandatory) measures of social isolation, social distancing, and hand washing were widely followed by the population (having generally mean values over 8.5 points in the scale), and the same trend was observed for the proxy variable “adherence to COVID-19 self-protective behaviors” that we had obtained by averaging the three previous scores. The statistical significance of the differences observed between the countries was corroborated by one-way ANOVA in the four variables: social isolation (F-statistic = 19.80, p-value < 0.001), social distancing (F-statistic = 16.81, p-value < 0.001), handwashing (F-statistic = 12.87, p-value < 0.001), and self-protective behaviors (F-statistic = 14.47, p-value < 0.001).

### Description of behavioral trends according to daily COVID-19 cases

**Fig 2:**
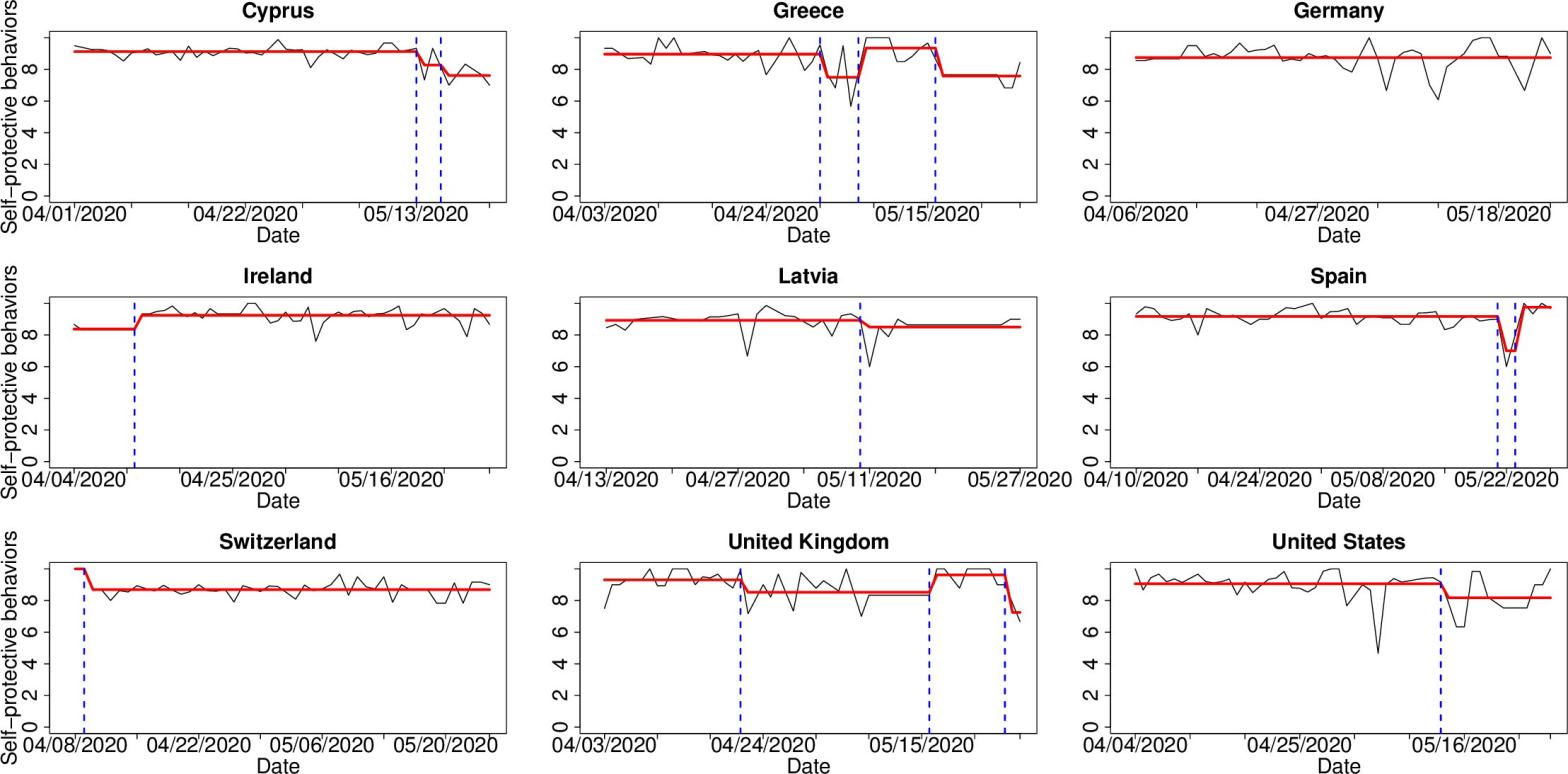
Results of the change-point detection analysis for the self-protective behaviors variable in nine countries. The real data are given with black colored line, while the estimated piecewise-constant signal is the red colored line. The estimated change-point locations are given with dotted, blue vertical lines.

The time series analysis allowed us to identify a relatively stable evolution in the follow-up of the public health authorities’ recommendations. However, although in general terms a high level of adherence was observed, the CPDA, using the Isolate-Detect methodology of Anastasiou and Fryzlewicz [30] allowed us to identify certain points that could indicate some abrupt changes in terms of adherence with the recommended behaviors (Fig 2).

**Fig 3:**
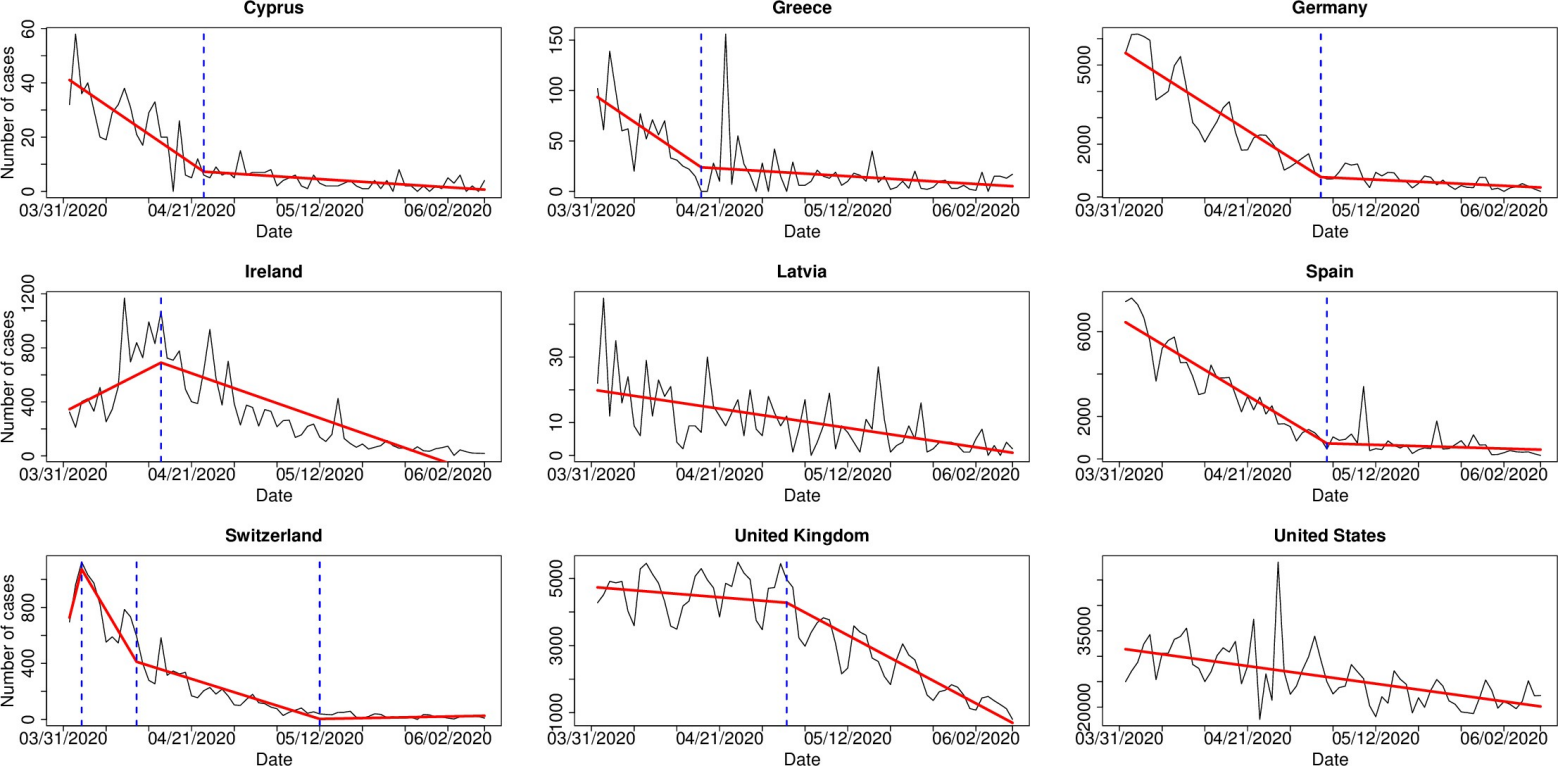
The real data (black colored line) and the estimated continuous, piecewise-linear signal (red colored line) for the daily number of COVID-19 confirmed cases in nine countries. The change-point locations are given with dotted, blue vertical lines.

As part of the interpretation of the detected change-points, their locations led us to consider possible factors that may have contributed to either the relaxation or the stricter adherence to self-protective behaviors of the populations living in the different countries that were affected–to a greater or lesser extent–by the COVID-19 pandemic. To this end, we ran a CPDA on the daily number of COVID-19 confirmed cases from the 1st of April 2020 until the 8th of June 2020 for all nine countries included in the study (Fig 3). Even though the impact of measures could be clearer in the daily number of COVID-19 related deaths rather than the daily number of confirmed cases (e. g. in [31]), in this manuscript we work on the number of cases due to the fact that for many countries in the study the daily number of deaths had been low (which could lead to quite unsafe conclusions if analyzed) during the period under study. To be more precise, during the 69 days under consideration, for Cyprus, Greece, Latvia, Spain, and Switzerland there had been 69, 47, 68, 12, 16 days, respectively, with at most 2 COVID-19 associated deaths. These are numbers that prevent a robust CPDA to be carried out with confidence.

With respect to Cyprus, we noticed that the prolonged period of a decrease on the daily number of cases, as shown in Fig 3, led to a relaxation of the adherence on the self-protective behaviors, which can be seen from Fig 2. We highlight that even though the only change-point detected for Cyprus in Fig 3 indicates a slight decrease on the magnitude of the strong negative trend, the latter still remains negative leading to the relaxation with respect to the self-protective behaviors. In the case of Greece, it is apparent from Fig 3, that the development of the pandemic in the country is extremely similar to the one in Cyprus. Furthermore, the last change-point for Greece in Fig 2 shows a relaxation of the adherence to self-protective behaviors, and this is in complete agreement to the two estimated change-points for Cyprus near mid-May. In Switzerland, the significant drop on the daily number of cases (apparent from the first change-point in Fig 3) seems to be directly connected to the first change-point (sudden drop in the self-protective behaviors) in Fig 2, showing that people seem to relax their adherence to preventive behaviors once there is a negative trend on the daily number of confirmed cases.

### ITSA using stringency of measures and daily cases

**Fig 4:**
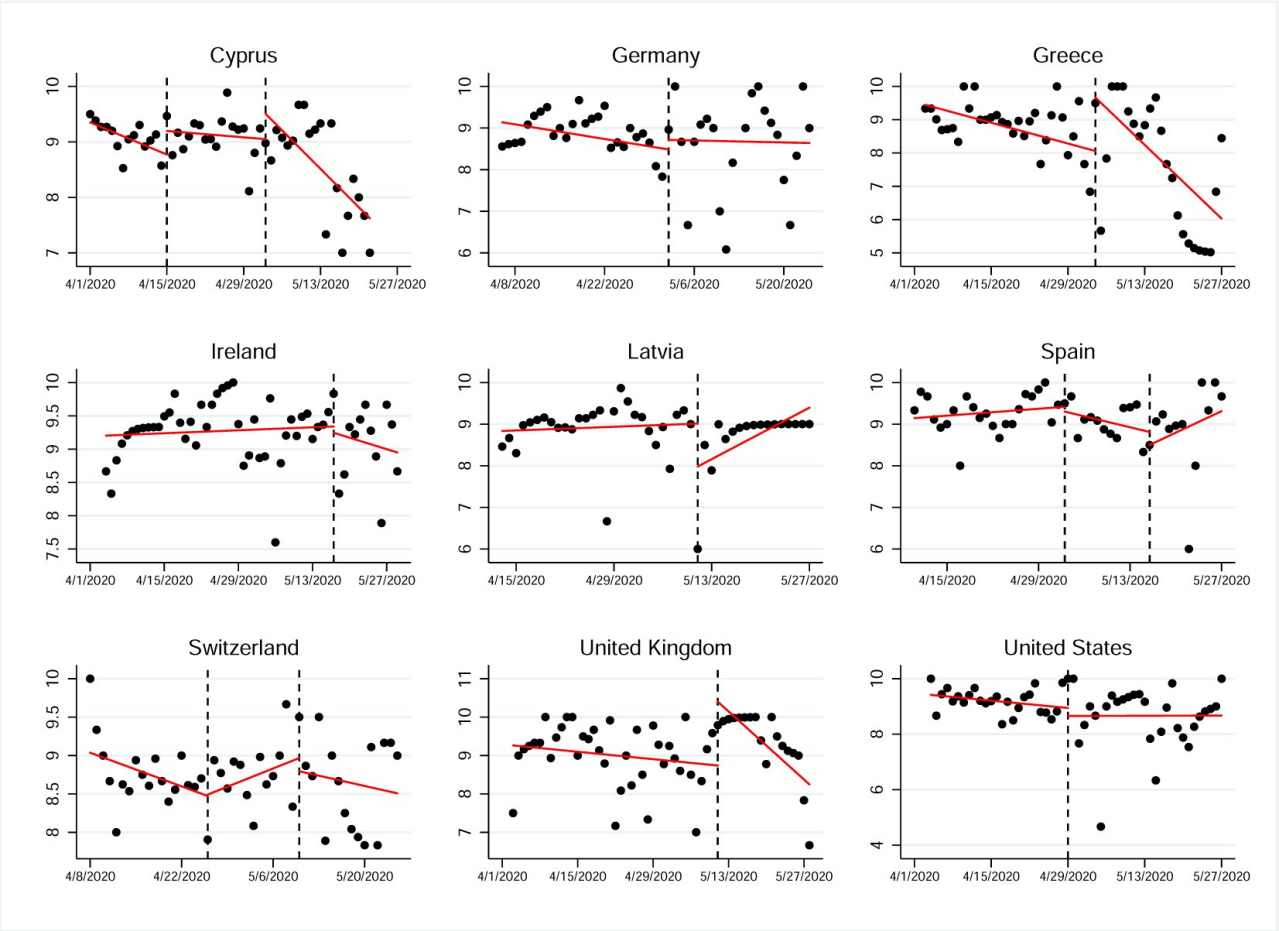
Interrupted time series analysis for self-protective behaviors using changes in stringency index as the intervention date (dashed horizontal line).

In order to assess the impact of governmental measures on the self-protective behaviors of the population, we used a stringency index to define the intervention dates in the ITSA. Specifically, what the intervention captured was the exact point of change in the level of stringency of the governmental measures applied to control the pandemic (Fig 4). Thus, the aim was to analyze whether the relaxation (or tightening) of the measures applied, would actually have an effect on the control of protective behaviors. In particular, it could be observed that the level of stress decreased (i.e., there was a relaxation in the measures applied) near the period when the population of countries such as Cyprus, Greece or the United Kingdom also relaxed their self-protective behaviors. However, this relaxation of behaviors did not occur in the rest of the countries that we analyzed. In fact, we found countries such as Germany, Ireland and the United States whose populations maintained their behaviors and others where they even increased their previous levels of protection, as was the case in Spain, Latvia and Switzerland (Table 1).

**Table 1:** Post-interventions linear trend with (decreasing) stringency index.

Country	Intervention	Coeff.	Std. Err.	t	P>t	[95% Conf.	Interval]
Cyprus	4/15/20	-0.008	0.020	-0.411	0.683	-0.049	0.032
Cyprus	5/3/20	-0.099***	0.022	-4.540	0.000	-0.143	-0.055
Germany	5/2/20	-0.003	0.048	-0.070	0.945	-0.100	0.093
Greece	5/4/20	-0.158**	0.071	-2.243	0.029	-0.300	-0.017
Ireland	5/17/20	-0.025	0.057	-0.432	0.668	-0.139	0.090
Latvia	5/11/20	0.088**	0.043	2.055	0.046	0.002	0.175
Spain	5/3/20	-0.025	0.024	-1.036	0.306	-0.074	0.024
Spain	5/18/20	0.176**	0.087	2.027	0.049	0.001	0.352
Switzerland	4/26/20	0.056*	0.033	1.717	0.093	-0.010	0.122
Switzerland	5/10/20	-0.010	0.030	-0.343	0.734	-0.070	0.050
United Kingdom	5/11/20	-0.127***	0.037	-3.393	0.001	-0.202	-0.052
United States	4/29/20	0.001	0.029	0.020	0.984	-0.059	0.060

Statistically significant coefficients at the level of 0.1*, 0.05**, and 0.01***

**Fig 5:**
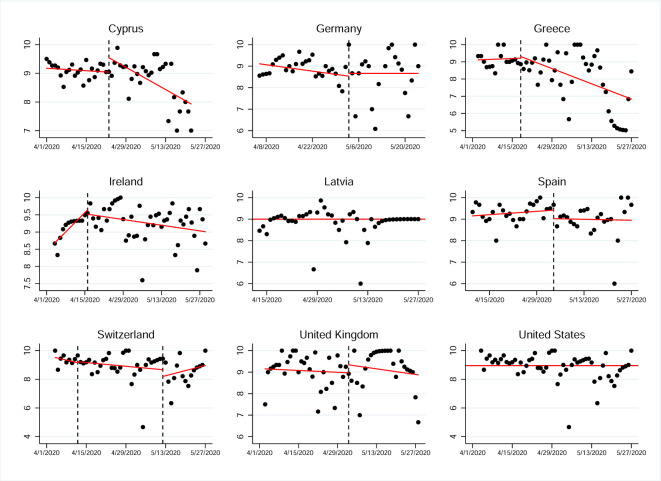
Interrupted time series analysis for self-protective behaviors using significant variation in COVID-19 cases as the intervention date (dashed horizontal line).

Subsequently, once the variations in protective behaviors had been analyzed in relation to changes in the stringency index, the variations in these behaviors would be studied, but in this case associated with changes in the number of cases (i.e., the CPDA to define the intervention dates). Using this variant, our aim was to infer the possible impact that the number of cases in a particular context (in this case, taking the country as the unit of reference) could have on the resulting protective behaviors. Trying to assess our second hypothesis, we wanted to observe how the perceived threat of the pandemic affected the control of protective behaviors. In other words, although we had been able to verify that the governmental measures applied to control the pandemic had not had the same impact on the populations in the different countries, our intention was now to understand the possible relationship of protective behaviors with the degree of the problem (based in the number of cases) that these same countries might have experienced (Fig 5).

As observed in the CPDA, when the daily number of COVID-19 confirmed cases started decreasing, people also started relaxing their protective behaviors (Cyprus, Greece, Ireland), although the opposite trend was observed in one country (Switzerland). For Latvia and the United States, we were unable to detect any significant variation in the daily number of cases that would allow us to define the intervention point for these countries during the period analyzed (Table 2).

**Table 2:** Post-interventions linear trend with (decreasing) COVID-19 cases.

Country	Intervention	Coeff.	Std. Err.	t	P>t	[95% Conf.	Interval]
Cyprus	4/23/20	-0.056***	0.015	-3.684	0.001	-0.086	-0.025
Germany	5/3/20	0.000	0.058	-0.003	0.998	-0.117	0.116
Greece	4/18/20	-0.063***	0.023	-2.704	0.009	-0.110	-0.016
Ireland	4/16/20	-0.012*	0.006	-1.944	0.057	-0.024	0.000
Spain	5/4/20	-0.003	0.030	-0.105	0.917	-0.064	0.058
Switzerland	4/12/20	0.014***	0.007	2.015	0.050	0.000	0.028
Switzerland	5/12/20	0.008	0.036	0.223	0.825	-0.065	0.082
United Kingdom	5/3/20	-0.019	0.034	-0.541	0.591	-0.088	0.050

Statistically significant coefficients at the level of 0.1*, 0.05**, and 0.01***

## Discussion

To our knowledge, this is the first study aimed at comparing the impact of government actions and risk perception on the promotion of self-protective behaviors during the COVID-19 pandemic using data from different countries. In this work, we initially assumed that government responses to control the spread of pandemic were able to reduce these behaviors (hypothesis 1), but we also hypothesized that, during health emergencies such as the COVID-19 pandemic, perceived threat could be a key self-controlling factor in the management of self-protective behaviors (hypothesis 2).

On the one hand, we found that the relaxation of governmental measures that aimed to prevent the spread of the disease also led to relaxation in population self-protective behaviors, particularly in countries such as Cyprus, Greece, and the United Kingdom. In this case, the parallelism between Cyprus and Greece could be due to the fact that the governments of these two geographically adjacent countries were in close consultation with each other regarding the treatment of the pandemic. However, other countries did not follow this pattern with even the opposite trend observed (e.g., Spain, Latvia, and Switzerland). In addition, the CPDA carried out showed that when the number of daily confirmed cases fell, people in some countries relaxed their self-protective behaviors (specifically, in Cyprus, Greece, and Switzerland), although this behavior was not consistently observed in all countries examined in this study. This is important since recent evidence from the UK showed that variation in adherence to self-protective behaviors can be higher when governmental rules change and therefore can be considered by policymakers when introducing new or changing existing guidelines [32]. Therefore, our two hypotheses were partially confirmed under certain country-specific conditions. In other words, we found that COVID-19 self-protective behaviors have been heterogeneous across different countries [33]. Inconsistent findings between countries may be attributed to diverse socio-demographic characteristics in samples collected for this study which can differently react to governmental guidelines and therefore adherence may depend on personal circumstances as well [34].

Pandemic fatigue could be considered as one of the different possible explanations in certain cases [35, 36] but this phenomenon cannot provide a single answer for all the countries that we analyzed nor was it specifically assessed in this study. For instance, the Spanish case is interesting considering that our analysis indicates that behaviors in Spain followed a trend contrary to the one expected. That is, when the Spanish government began to relax prevention measures, self-protective behaviors increased in the country. This may mean that the population behavior is more closely related to the severity of the pandemic in that country rather than governmental responses. Moreover, when people were isolating during lockdown, they probably needed to engage less in the self-protective behaviors but when the restrictions were lifted, they needed to become more vigilant and engage in more protection. In other words, we could hypothesize the level of the health emergency and its association with the perceived risk and fear of the population might be a stronger determinant of self-protective behaviors. Indeed, as a recent study has shown, the Spanish adult population may have adapted to the new pandemic context by progressively improving their health behaviors [37]. Therefore, the increase in COVID-19 protective behaviors after the first few months of the pandemic in this country could be linked to increased hazard perception once the first wave of the disease has passed, which leads us to conclude that the perceived sense of risk at the population level may have a greater impact on collective behaviors than government-directed changes at the regulatory level.

How populations changed their behavior during the COVID-19 pandemic can also be an artifact of their response to adjustments in risk assessment since risk perceptions seem to influence COVID-19 protective behaviors similarly to how they impact other health outcomes [38]. Monitoring how the news and information on cases and deaths at different countries are spread and presented to the public can benefit public health to prevent propensity to act in a riskless manner by reducing adherence to protective behaviors. Previous research has been limited into looking at how governmental stringency measures influence population behaviors which we have been able to demonstrate using the stringency index and estimate the enforcement’s explanatory power on adherence changes over time. It can be that public health messages can highlight citizen’s collaborative role in combating the long-term impact of the COVID-19 pandemic as demonstrated by evidence from the USA and Finland recently [39].

We acknowledge several limitations. First, our study is based on a time period that is relatively short considering the temporal breadth of the pandemic and with data from the early stages of the COVID-19 pandemic. In addition, it is necessary to consider that along this period many countries were incorporated progressively, which would result in different sample sizes. Secondly, although the initial sample included 77 countries, for the present study we only selected those countries with an adequate sample size to be able to work with an aggregation of the data at a temporal level. Furthermore, because of the fact that we did not have access to direct measures of fluctuations in risk perception, we considered the trend for the daily number of confirmed COVID-19 cases as informational causes of perceived threat. Finally, and in line with the previous limitation, since our objective was to trace collective behaviors in relation to governmental protective measures and the number of confirmed COVID-19 cases, our conclusions can only be extrapolated at the aggregate level, so that we cannot draw individual conclusions on the behaviors of different profiles of individuals. Future studies can also investigate differences across behaviors since changes in adherence can be contingent on type of behavior [33].

Despite the aforementioned limitations this study presents the methodological novelty of combining a data-based approach with another approach based on previous evidence. Thus, the CPDA technique has allowed us to automatically establish variations in the pandemic whose effect would later be assessed by means of ITSA, which has allowed us to identify differences in self-protection behaviors associated with country-specific events. Therefore, this paper presents how a data-driven approach can be combined with an evidence-based approach through two different time-series techniques (i.e., change point detection analysis and interrupted time series analysis). This combination of analytical approaches has allowed us to describe variations in self-protection behaviors in different geographical contexts, as well as to determine and compare the impact that governmental interventions and risk perceptions may have had on the course of the pandemic in individuals from different countries. By using these techniques, we have been able to verify that the control measures applied for the promotion of self-protective behaviors have not been equally effective in all countries, and that not all countries have responded similarly to the evolution of the pandemic.

## Conclusion

Our findings indicate that the promotion of self-protective behaviors should be tailored to the specific (pandemic) circumstances of the country in which such measures are to be applied, as different public health interventions may be received differently depending on the specific context and, specifically, according to the population’s risk perceptions. In addition, monitoring how the news and information regarding the COVID-19 cases and deaths in different countries are spread and presented to the public could benefit government and health agencies to prevent population propensity to relaxing self-protective behaviors.
